# Case Report: A Case of Leukocyte Adhesion Deficiency, Type III Presenting With Impaired Platelet Function, Lymphocytosis and Granulocytosis

**DOI:** 10.3389/fped.2021.713921

**Published:** 2021-08-16

**Authors:** Amal M. Yahya, Asia A. AlMulla, Haydar J. AlRufaye, Ahmed Al Dhaheri, Abdulghani S. Elomami, Suleiman Al-Hammadi, Lalitha Kailas, Ranjit Vijayan, Abdul-Kader Souid

**Affiliations:** ^1^Department of Pediatrics, Tawam Hospital, Al Ain, United Arab Emirates; ^2^Department of Hematology-Oncology, Tawam Hospital, Al Ain, United Arab Emirates; ^3^Department of Pathology, Tawam Hospital, Al Ain, United Arab Emirates; ^4^College of Medicine, Mohamed Bin Rashid University of Medicine and Health Sciences, Dubai, United Arab Emirates; ^5^Department of Pediatrics, College of Medicine and Health Sciences, United Arab Emirates University, Al Ain, United Arab Emirates; ^6^Department of Pediatrics, Sree Gokulam Medical College, Thiruvananthapuram, India; ^7^Department of Biology, College of Science, United Arab Emirates University, Al Ain, United Arab Emirates

**Keywords:** platelet dysfunction, epistaxis, Glanzmann thrombasthenia, leukocytosis, inborn error of immunity, BCG vaccine, FERMT3, kindlin-3

## Abstract

Fermitin family homolog 3 (FERMT3), alternatively kindlin-3 (KIND3), is an integrin binding protein (of 667 residues) encoded by the *FERMT3* gene. The molecule is essential for activating integrin α_IIb_β_3_ (the fibrinogen receptor) on platelets and for the integrin-mediated hematopoietic cell (including platelets, T lymphocytes, B lymphocytes, and granulocytes) adhesion. Its defects are associated with impaired primary hemostasis, described as “Glanzmann's thrombasthenia (MIM#273800)-like bleeding problem.” The defects are also associated with infections, designated as “LAD1 (leukocyte adhesion deficiency, type I; MIM#116920)-like immune deficiency.” The entity that joins the impaired primary hemostasis with the leukocyte malfunction has been termed “leukocyte adhesion deficiency, type III” (LAD3, autosomal recessive, MIM#612840), representing a defective activation of the integrins β_1_, β_2_, and β3 on leukocytes and platelets. Here, we report a male toddler with novel compound heterozygous variants, NM_178443.2(*FERMT3*):c.1800G>A, p.Trp600^*^ (a non-sense variant) and NM_178443.2(*FERMT3*):c.2001del p.^*^668Glufs^*^106 (a non-stop variant). His umbilical cord separated at about 3 weeks of age. A skin rash (mainly petechiae and purpura) and recurrent episodes of severe epistaxis required blood transfusions in early infancy. His hemostatic work-up was remarkable for a normal platelet count, but abnormal platelet function screen with markedly prolonged collagen-epinephrine and collagen-ADP closure times. The impaired platelet function was associated with reduced platelet aggregation with all agonists. The expression of platelet receptors was normal. Other remarkable findings were persistent lymphocytosis and granulocytosis, representing defects in diapedesis due to the integrin dysfunction. The natural history of his condition, structure and sequence analysis of the variations, and comparison with other LAD3 cases reported in the literature are presented.

## Introduction

The kindlin family member 3 [kindlin-3, or KIND3; preferred name FERMT3 (Uniprot accession Q86UX7)] is a cytosolic “β-integrin adapter protein” expressed mainly in hematopoietic cells (1, 2). Deficiencies in FERMT3 have been linked to the autosomal recessive “leukocyte adhesion deficiency, type III” (LAD3, MIM#612840), where all the β-integrins (β_1_, β_2_, and β_3_) are defective (3–8). This entity is a joint of LAD1-like leukocyte dysfunction (persistent leukocytosis plus infections, typically without pus formation) and Glanzmann thrombasthenia-like platelet dysfunction (mucocutaneous bleeding) (9). Individuals with this condition present in early infancy with severe bleeding and frequent infections (10).

The FERM central domain (residues 258 to 558; InterPro accession: Q86UX7) of FERMT3 binds to and activates platelet integrin α_IIb_β_3_ (CD41/CD61). This receptor is activated by ADP, epinephrine, collagen, arachidonic acid, and thrombin. Activated receptors then bind fibrinogen, von Willebrand factor (vWF), fibronectin, and vitronectin (11–13). FERM domain is also essential for activating integrins β_1_ and β_2_ on the surface of granulocytes and lymphocytes (14–16). FERM defects, thus, demonstrate the essential role of F-actin-rich structures in the podosomes of lymphocytes, granulocytes and platelets (17, 18).

Only very few children with LAD3 have been reported in the literature. Thus, clinical descriptions of new patients are important to define the disease phenotype. We present here a toddler with novel compound heterozygous variants in *FERMT3* (MIM#607901). This report features his characteristics and natural history. Its purpose is to enhance the recognition of this disorder and perform a structural bioinformatics analysis of his variations.

## Case Report

This 18-month-old male toddler was born at term to asymptomatic, non-consanguineous parents (from Kerala, India). The pregnancy and delivery were uneventful. His birth weight was 2.9 kg. He had no siblings; the mother was gravida 2, para 1, abortion (spontaneous) 1. His paternal grandmother had severe menorrhagia, requiring multiple transfusions. Otherwise, the family history was negative.

**Figure 1:**
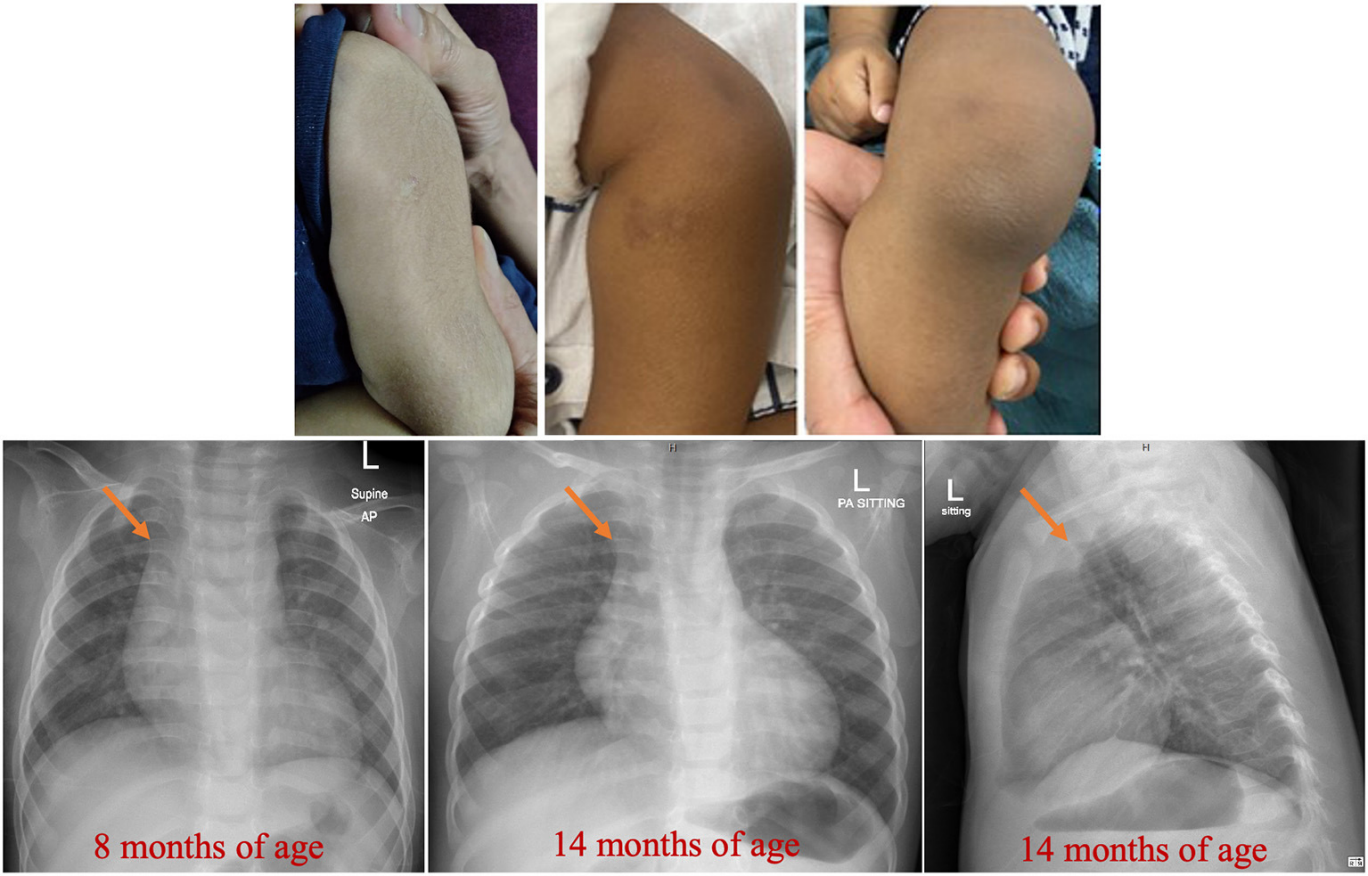
Findings on the skin and chest radiographs. Healed BCG site and dry skin with bruises and scars are evident. Prominent thymus (arrowed) at 8 mo of age is evident. Please also note the normal skeletal radiographs.

He was born in the United Arab Emirates and received the BCG (Bacillus Calmette–Guérin) vaccine [0.05 mL, intradermal injection in the upper left arm (manufactured by Serum Institute of India Pvt. Ltd., India)] at birth (Figure 1, left upper panel). His postnatal period was complicated by a delayed umbilical cord separation for about 3 weeks. He presented at 2 months of age with petechial rash over his arms and seborrheic dermatitis over his scalp. Starting at 8 months of age, he had recurrent episodes of severe nose bleeding; consequently, several blood counts were requested. He had 10 admissions since birth; six for severe epistaxis, two for work-up of marked leukocytosis, one for the bone marrow procedure, and one for a significant skin rash (eczema-like). His overall management included two red cell transfusions (over a 4 month period), three platelet transfusions, and tranexamic acid (used only for a short time period).

He was never febrile; his temperatures were ≤37.2°C. On two occasions, his blood cultures (requested because of the marked leukocytosis) grew *Micrococcus luteus* and *Staphylococcus haemolyticus*, which were considered skin flora contaminants. His physical examination was remarkable for the skin findings of dryness, bruises (petechiae and purpura), and a few scars, as shown in Figure 1 (middle and right upper panel). He was uncircumcised. His growth and development were appropriate for age.

**Figure 2:**
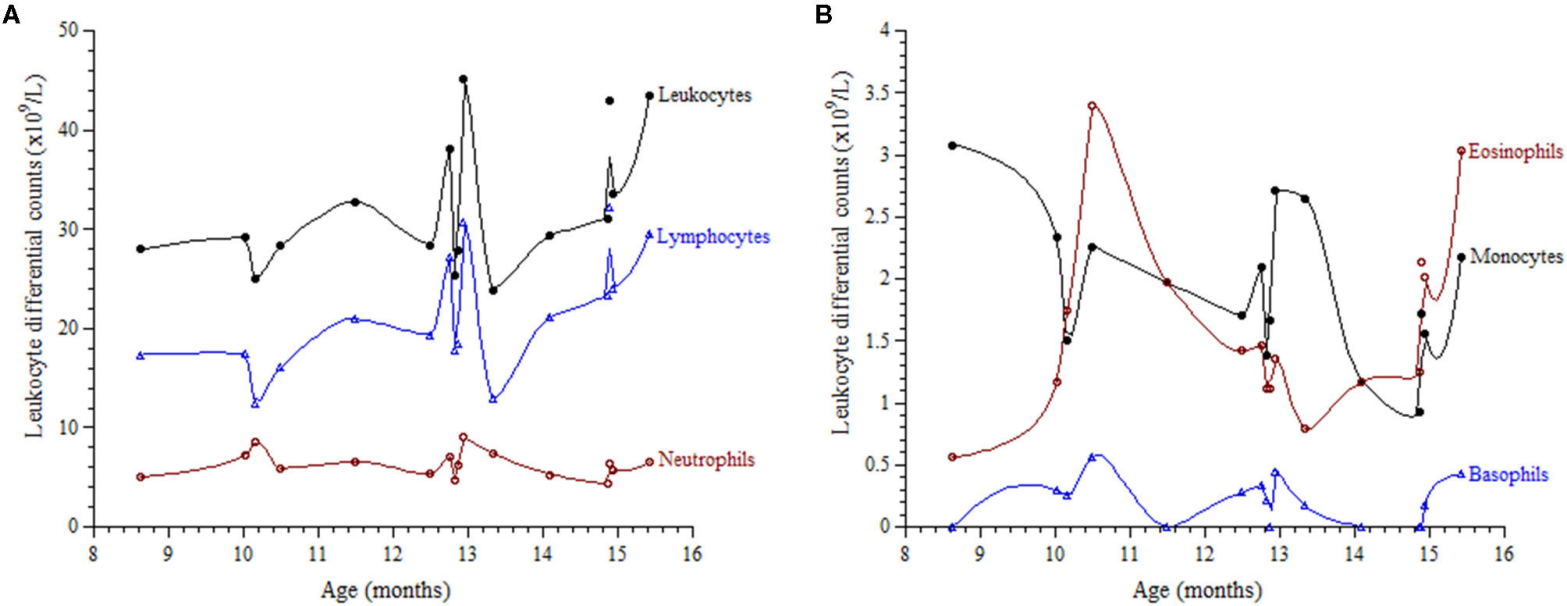
Leukocyte differential counts as function of age.

**Figure 3:**
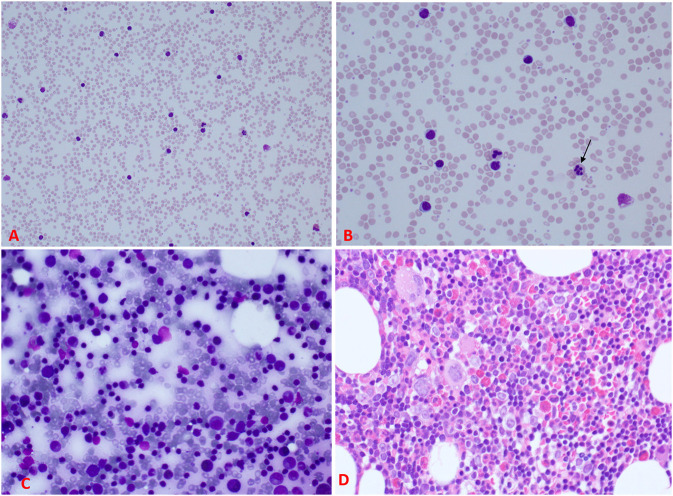
Peripheral blood and bone marrow morphology. **(A)** A low power view of the peripheral blood smear demonstrating significant leukocytosis. **(B)** A higher magnification of the smear showing the leukocytosis predominantly lymphocytes mixed with neutrophils; some of the neutrophils reveal abnormal nuclear lobation, like hypersegmentation (arrow). **(C)** Bone marrow aspirate showing normal maturation of erythroid and myeloid cells and increased lymphocytes with some immaturity suggestive of hematogones. **(D)** Section from the bone marrow biopsy showing about 70% cellularity (normal for age) of trilineage hematopoiesis with increased eosinophils, lymphocyte aggregates, and interstitial lymphocytes. Megakaryocytes were adequate with normal morphology [For comparison, please see (19) for normal peripheral blood and bone marrow smears].

Investigations were requested because of the unexplained persistent leukocytosis. His white blood cell differential counts are shown in Figures 2A,B. Persistent marked leukocytosis (mainly lymphocytosis, monocytosis, and eosinophilia) were evident. In addition, “left shift” (neutrophil bands, metamyelocytes, and myelocytes) was a frequent finding (Figures 3A,B). His chest radiograph was remarkable for a *notably* prominent thymus (reported as a “round opacity at the mediastinum”), Figure 1 (lower panels). Findings on the abdominal ultrasound and echocardiogram were normal. Serum ferritin was ≤20 μg/L and C-reactive protein (CRP) ≤ 1.8 mg/L. Serum immunoglobulins (IgA, IgG, and IgM) were normal. At 9 months of age, his lymphocyte subset was: CD3 T cells [11,002/μL (41%), reference values (10th−90th centiles) = 1,900–5,900], CD4 T cells [6,423/μL (24%), reference values = 1,400–4,300], CD8 T cells [3,785/μL (14%), reference values = 500–1,700], CD19 B cells [14,206/μL (53%), reference values = 610–2,600], and NK cells (426/μL, 2%). Bone marrow examination was normal; the megakaryocytes were adequate in number and had normal morphology (Figures 3C,D). Leukocyte RNA was negative for leukemia fusion gene transcripts (MDx Leukemia Fusion Gene Q30 screen; QuanDx, Menlo Park, CA, USA).

Hemostatic evaluation was also requested because of the severe epistaxis. His platelet counts and secondary hemostasis [activated partial thromboplastin time (aPTT), prothrombin time (PT), international normalized ratio (INR), thrombin time (TT), and serum fibrinogen] were normal. Platelet function screen (performed using Dade® PFA Collagen/EPI and Collagen/ADP Test Cartridges, Siemens Healthcare Diagnostics GmbH; Marburg, Germany) showed prolonged collagen-epinephrine and collagen-ADP closure times > 300 s (normal, ≤ 164 s). Von Willebrand factor (vWF) antigen was 59.6% (normal, 50–200) and activity 46.7% (normal, 50–200); these tests were performed using Innovance® VWF Ac and VWF Ag (Siemens Healthcare Diagnostics GmbH; Marburg, Germany). The results of vWF were normal as his blood group was “O”; even though, the red blood cell phenotype may have partially contributed to his bleeding tendency. Platelet aggregation studies (performed using the Bio/Data Corporation product; Horsham, PA, USA) revealed severely impaired aggregations in response to all agonists, suggesting a defect in the inside-out activation of integrin α_IIb_β_3_ (Table 1). Spontaneous platelet aggregation, a pathologic finding, was not present.

**Table 1:** Results of his platelet aggregation studies.

	**Proband**	**Normal control**
**Ristocetin**		
1.5 mg/mL	15%	>50
1.0 mg/mL	7%	>50
0.5 mg/mL	4%	4%
**Thrombin**		
1.0 U/mL	23%	>50
**ADP**		
10 μmol/L	4%	>50
5 μmol/L	4%	>50
2.5 μmol/L	4%	>50
1.25 μmol/L	4%	>50
**Epinephrine**		
10 μmol/L	4%	>50
5 μmol/L	4%	>50
**Arachidonic acid**		
500 μg/mL	4%	>50
250 μg/mL	3%	>50
**Collagen**		
2 μg/mL	10%	>50
1 μg/mL	9%	>50

Flow cytometry revealed a normal expression of the leukocyte receptors CD11a (integrin, alpha L), CD11b (integrin, alpha M), CD11c (integrin, alpha X), and CD18 (integrin β_2_). It also revealed a normal expression (99%) of the platelet receptors CD41 (integrin α_IIb_), CD61 (integrin β_3_), CD42a (GPIX), and CD42b (GPlb-α). These tests were performed using monoclonal antibodies from Becton, Dickinson and Company, BD Biosciences; San Jose, California, USA (see Supplementary Material).

In view of his abnormal platelet function profile and marked leukocytosis, “diagnostic exome sequencing test” was requested (mainly considering the probability of LAD3). Two novel compound heterozygous variants were found: (1) Non-sense (stop-gain, resulting in a truncated product) NM_178443.2(*FERMT3*):c.1800G>A, p.Trp600^*^ (inherited from the mother), and (2) Non-stop (stop-loss, resulting in an elongated polypeptide) NM_178443.2(*FERMT3*):c.2001del p.^*^668Glufs^*^106 (inherited from the father).

**Figure 4:**
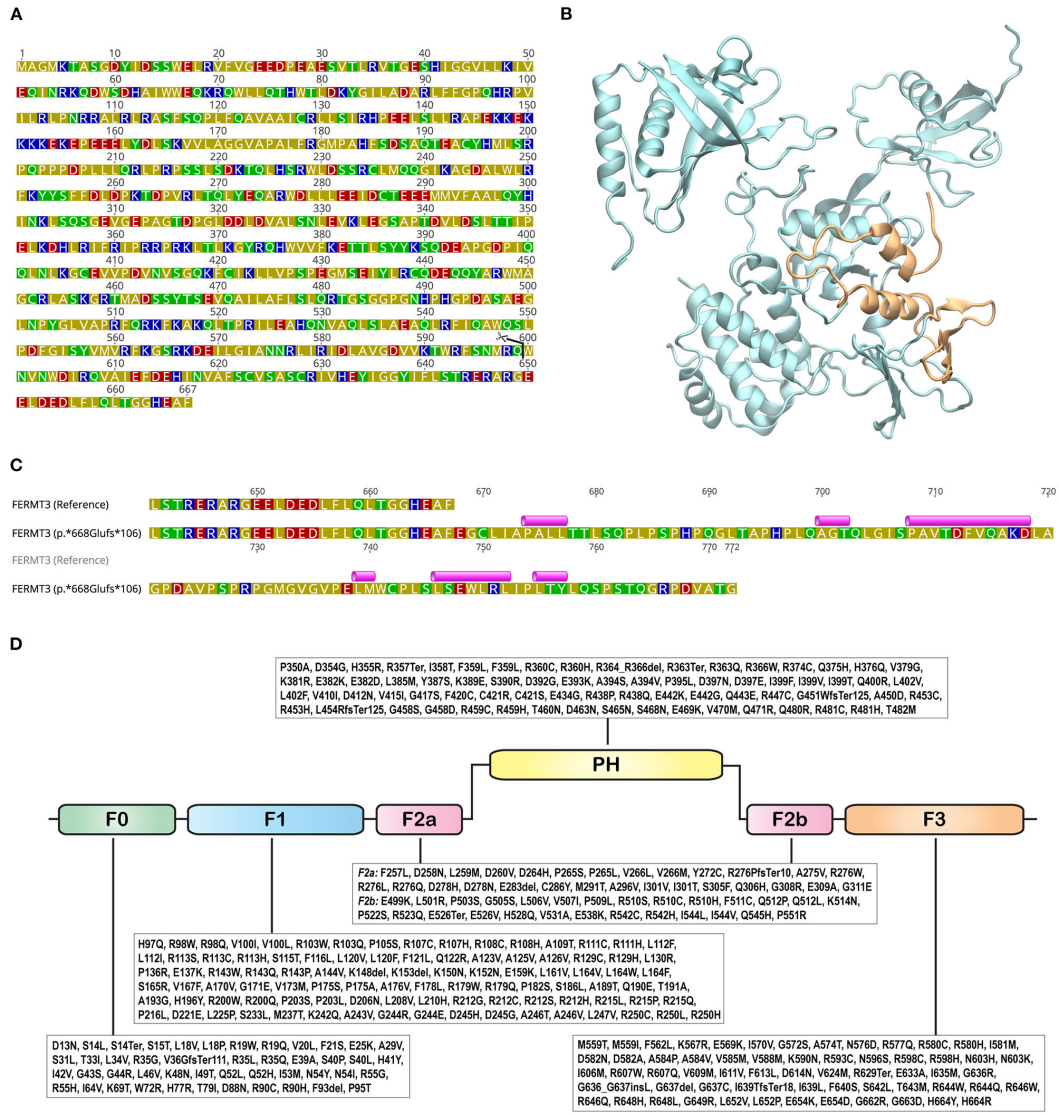
Structure and sequence of FERMT3. **(A)** Sequence of FERMT3 (NCBI Accession: NP_848537.1) with the site of truncation in p.Trp600* shown with a pair of scissors. Amino acids are colored based on their polarity: blue - basic; red - acidic; green - polar; olive – hydrophobic. **(B)** Three dimensional protein structure of FERMT3, generated using the Protein Data Bank structure 7C3M, depicted in cartoon representation. The region truncated in the p.Trp600* non-sense variant is shown in orange. **(C)** Protein sequence alignment of FERMT3 (NCBI Accession: NP_848537.1) and the p.*668Glufs*106 non-stop variant starting from position 640. Secondary structures, predicted using PSIPRED 4.0 (Predict Secondary Structure; http://bioinf.cs.ucl.ac.uk/psipred/), in the extended region of the variant are shown above the sequence. Violet cylinders represent α-helices. **(D)** Variations identified in the canonical Ensembl transcript (ENST00000279227.5) were from gnomAD v2.1.1 are shown with amino acids represented by their one letter codes. FERM domains (sub-domains F0, F1, F2a, F2b, and F3; InterPro classifies region 258-558 as FERM central domain) and pleckstrin homology (PH) are shown as rounded rectangles.

The non-sense variant *FERMT3*:p.Trp600^*^ leads to a premature truncation of the protein and the loss of 68 residues from the C-terminal (Figure 4A). This truncation results in the loss of two α-helices and a β-sheet, comprising of three β-strands (Figure 4B). Computational prediction scores from multiple predictors [combined annotation dependent depletion (CADD): 46; likelihood ratio test (LRT): 0.8433; MutationTaster: 0.81] obtained from Ensembl Variant Effect Predictor (VEP; https://www.ensembl.org/Tools/VEP) (20) suggest that this variant is “deleterious,” while Varsome (21) flags it as “pathogenic.” The Trp600^*^ variation shortens the F3 subdomain of FERMT3. The F3 subdomain is a part of the integrin-binding pocket of FERMT3 and this variation is, therefore, expected to inhibit the ability of FERMT3 to bind to integrins.

The p.^*^668Glufs^*^106 non-stop variant results in an extended polypeptide with an additional 105 amino acids, a 16% extension to the wild-type sequence. Basic Local Alignment Search Tool (BLAST; https://blast.ncbi.nlm.nih.gov) (22) searches did not identify any homologous sequences or structures. Secondary structure prediction with PSIPRED (http://bioinf.cs.ucl.ac.uk/psipred/) (23) indicated that this region could potentially harbor six α-helices (Figure 4C). While secondary structures have been predicted in the extended sequence of p.^*^668Glufs^*^106 (Figure 4C), it is unclear if this extended part will cooperatively fold to produce a functional domain. Nonetheless, even if it does, its close proximity to the integrin-binding pocket of the F3 subdomain, located near the C-terminal of the protein, is likely to disrupt its ability to bind to integrins.

## Discussion

Pathogenic variants of *FERMT3* (MIM#607901) cause LAD3 (MIM#612840). GnomAD v2.1.1 lists 1,112 variations in *FERMT3*^1^. Of these, 372 are start-loss, stop-loss, inframe deletion, inframe insertion, frameshift, or non-sense mutations (6, 24). Variations that map on to the FERM (F: 4.1 protein, E: ezrin, R: radixin, and M: moesin) and PH (pleckstrin homology) domains of FERMT3 are shown in Figure 4D.

As indicated above, LAD3 is joint defects of LAD1 (MIM#116920)-like immune deficiency and Glanzmann thrombasthenia (MIM#273800)-like bleeding. The defect involves the intracellular protein that interacts with β-integrins in hematopoietic cells, including granulocytes, lymphocytes, macrophages, and platelets. The predominant phenotypes result from failures to activate β_1_ and β_2_ integrins on the surface of granulocytes and lymphocytes and β_3_ integrin (α_IIb_β_3_) on the surface of platelets (13, 14). As previously shown, integrins α_2_β_1_ and α_IIb_β_3_ support the irreversible adhesive interactions of platelets with collagen and VWF; these receptors also support the platelet recruitment and spreading (25). Integrin β_1_ (CD29) also pairs with other α subunits, such as α_4_β_1_ (a vascular cell adhesion protein 1, VCAM-1, receptor), α_5_β_1_ (a fibronectin receptor), and α_6_β_1_ (a laminin receptor) (26). Integrin β_2_ (CD18), on the other hand, is exclusively expressed on the surface of leukocytes. Its pairing with one of a number of distinct α subunits (e.g., α_M_, or CD11b) enables cell-cell interactions, such as the binding to intercellular adhesion molecule 1 (ICAM-1) on an activated endothelial cell thus mediating neutrophil adhesion and spreading (27). Similarly, integrin β_3_ (CD61) was shown to pair with the α_v_ subunit; the resulting receptor (α_v_β_3_) binds the secreted protein vitronectin and mediates cell adhesion, spreading and signaling (28). Defective “inside-to-outside” signalings explain the hallmarks of the entity of granulocytosis (innate immune defects), lymphocytosis (defective adaptive immunity) and life-threatening mucocutaneous bleeding (impaired primary hemostasis) (14, 15, 29, 30).

Noticeable features of his phenotype (see reference 8, and **Table 3**) include: (1) Delayed umbilical cord separation for about 3 weeks (reference mean ± SD time = 6.61 ± 2.33 days; confidence of interval 95% = 6.16–7.05) (31); (2) Spontaneous petechiae in the neonatal period; (3) Severe epistaxis in early infancy (his first episode was at 8 mo of age, requiring transfusion); (4) Normal secondary hemostasis; (5) Normal platelet count; (6) Abnormal platelet function screen (PFA-100® test, >300 s for both channels); (7) Normal von Willebrand factor antigen and activity; (8) Impaired platelet aggregation with all agonists (ristocetin, thrombin, ADP, epinephrine, arachidonic acid, and collagen); (9) Normal expression of platelet receptors (e.g., CD41/CD61, or integrin α_IIb_β_3_); (10) Normal expression of leukocyte adhesion molecules (e.g., CD18, or integrin β_2_); (11) Absent hepatosplenomegaly and lymphadenopathy; (12) Prominent thymus on the chest radiograph; (13) Absence of fever; (14) Normal inflammatory biomarkers (CRP and ferritin); (15) Persistent marked leukocytosis/ granulocytosis (most notably, high monocyte and eosinophil counts); and (16) Persistent lymphocytosis (high T cell and B cell counts; with C19 B cell count higher than CD3 T cell count).

It is worth emphasizing that the recurrent severe episodes of epistaxis were the foremost clinical manifestation. This problem triggered the extensive hemostatic evaluation and genetic testing. In our community, diagnostic exome sequencing provides about 50% yield in detecting inborn errors metabolism (36). Thus, this test was requested for his investigation. It is also worth noting that his hemostatic findings suggested variants in *FERMT3* (see below).

The differential diagnosis of leukocytosis includes infection, leukemoid reaction [leukocytosis with circulating immature white and red blood cells (“left shift”), mimicking chronic myelomonocytic leukemia (CMML) and chronic myelogenous leukemia (CML)], and lymphoproliferation (including leukemia) (37). The eosinophilia may mimic atopy. The large thymus may mimic T cell lymphoma. Nevertheless, persistent marked elevations of granulocytes and lymphocytes suggest a leukocyte adhesion defect (8).

The differential diagnosis of impaired primary hemostasis (summarized in Table 2) includes von Willebrand disease, acquired platelet dysfunction (e.g., use of non-steroidal anti-inflammatory drugs), inherited platelet dysfunction [e.g., Glanzmann thrombasthenia (GT), Bernard-Soulier syndrome (BSS), ADP receptor defect, and gray platelet syndrome], and vascular anomaly (e.g., Ehlers-Danlos syndrome) (37). It is important to note, Glanzmann thrombasthenia is characterized by a diminished platelet aggregation with ADP, epinephrine, collagen, thrombin, and arachidonic acid, with normal agglutination with ristocetin. Bernard-Soulier syndrome, on the other hand, is associated with normal platelet aggregation with all agonists, except ristocetin. Diminished platelet aggregation in response to ADP alone is characteristic of ADP receptor defect. In gray platelet syndrome, platelet aggregation is variable and can be normal; however, thrombocytopenia is typically present (38).

**Table 2:** Schema diagram summarizing the hemostatic assessment.

*I. Basic hemostatic work-up* Platelet count Platelet function screen von Willebrand factor (vWF) antigen and activity aPTT, PT/INR, thrombin time, and fibrinogen
*II. Platelet aggregation studies (for an abnormal platelet function screen with normal vWF)* Abnormal agglutination with “ristocetin” only → BSS Abnormal aggregation with “thrombin, ADP, collagen, arachidonic acid and epinephrine” → GT Abnormal aggregation with “ADP” only → ADP defect Abnormal aggregation with all agonists → LAD3
*III. Platelet receptor expression (for abnormal platelet aggregation studies)* Reduced expression of “CD42a (GPIX) and CD42b (GPlb-α)” → BSS Reduced expression of “CD41 (integrin α_IIb_) and CD61 (integrin β_3_)” → GT Normal expression of all receptors → LAD3

Defects in natural killer cell migration, adhesion and activation have been also reported in LAD3 (39). Thus, this entity represents a multifaceted inborn error of immunity. This consideration is especially important for countries which implement universal BCG vaccination at birth, such as India and United Arab Emirates. Furthermore, defects in bone-resorbing osteoclast adhesion have been reported in LAD3 (32, 40), justifying its inclusion in the differential diagnosis of impaired bone homeostasis (8).

The expediency of his abnormal “*platelet function screen*” is evident. This test assesses primary hemostasis when the platelet count is normal, and has replaced the old “bleeding time.” Thus, the platelet function screen test is essential in the current hemostatic evaluation (41).

Treatment of LAD3 is supportive and includes topical thrombin in conjunction with an anti-fibrinolytic agent, tranexamic acid or aminocaproic acid. The response to anti-fibrinolytic agents should be observed, and the dose should be adjusted as necessary. Platelet transfusion is indicated in severe bleeding, or it can be administered weekly as prophylaxis when necessary (8). Precautions with regard to circumcision and skin and mucosal (including dental) hygiene are necessary. Prompt antimicrobial therapy in case of infection is also vital, particularly as fever and inflammatory biomarkers may not be present or prominent (42). Prophylaxis for pneumocystis pneumonia (typically with the use of trimethoprim/sulfamethoxazole) is needed (see Table 3).

**Table 3:** Natural histories of the current and previously reported *FERMT3* (Ensemble transcript: ENST00000279227.5) variants.

**Variants/Cases**	**Age at presentation (mo)**	**Age at diagnosis (mo)**	**Bleeding**	**Lymphocytosis**	**Granulocytosis**	**Infections**	**Organisms**	**Management**	**References**
p.Trp600* (Pathogenic)/ p.*668Glufs*106	3	10	+	+	+	–		Platelet and blood transfusions; tranexamic acid	Current study
p.Gln599Pro(Uncertain significance)Homozygous	3	5	+	+	+	+	*Acinetobacter baumannii, Salmonella species, Pseudomonas aeruginosa*	Immunoglobulin infusions (for low serum IgG); antibiotics	(3)
p.Trp16*(rs121918296; Pathogenic) Homozygous	0.5		+	+	+	+		Platelet and blood transfusions; antibiotics; bone marrow transplant	(5)
p.Gln96*(Pathogenic)Homozygous	4	7	+		+	+	*Burkholderia cepacia*	Blood and platelet transfusions; antibiotics	(6)
p.Arg357*(rs1354176121; Pathogenic)Homozygous	0.25	1	+		+	+	*Candida albicans, Pseudomonas aeruginosa, Staphylococcus aureus*	Platelet and plasma transfusions; antibiotics; colostomy; bone marrow transplant	(7)
p. Gly308Arg/ p.Pro429ArgAla*	0	11	+		+	+	*Pneumocystis jirovecii, Pseudomonas aeruginosa*	Bone marrow transplant	(15)
p.Gln533*(Pathogenic) Homozygous	0.25		+		+	+	*Pseudomonas aeruginosa*	Platelet and blood transfusions; antibiotics; bone marrow transplant (at 5 mo of age)	(32)
p.Gln480*(Pathogenic)Homozygous	0.75		+		+	+	*Candida albicans, Staphylococcus aureus*	Bone marrow transplant at 8 mo of age	(33)
p.Asn54Argfs*142Homozygous	At birth		+	+	+	+	*Pneumocystis jirovecii*	Immunoglobulin infusions (for low serum IgG); antibiotics	(34)
Five children;3 missense variants and one nonsense variant	0.8(median)	34(median)	+	+	+	Infections in 1/5 patients	Omphalitis in 3/5 patients		(24)
Six children:p.Arg513* (3 children; rs121918295; Pathogenic); p.Arg577* (rs121918297; Pathogenic), p.Lys82Thrfs*67, p.Asp397Thrfs*29			+	+	+	+			(35)

*Pathogenicity is based on Varsome*.

In one report, platelet and granulocyte functions (including leukocytosis) and radiographic signs of osteosclerosis (osteopetrosis) were normalized after allogeneic hematopoietic stem cell transplantation (8). Thus, this therapeutic intervention is successful, especially if applied in infancy (43–46). Of note, our patient had no radiographic signs of osteopetrosis, as shown in Figure 1. Genetic prevention remains a foreseen goal.

As noted above, he had received the live vaccine BCG (Bacillus Calmette–Guérin). Leucocyte adhesion deficiencies are included in the list of inborn errors of immunity (IEI) that describes complications to the BCG vaccination in India (47). In a retrospective study, inborn errors of immunity were identified in 52 of 90 (58%) individuals who had a localized or disseminated BCG complication; these disorders included LAD1 (47). Although he had no evidence of BCG or TB (tuberculosis) disease, these complications (which are generally severe in infants) should be considered, especially in high TB burden countries such as India. Therefore, LAD3 should be added to the list of “defects in intrinsic and innate immunity” where BCG should be avoided as a precaution, and a prophylactic treatment for BCG disease should be considered (48, 49). Moreover, it is prudent to change the time of BCG administration from birth to 1 year of age, when most IEI become more recognizable (50). It is well to reemphasize that a cautious consideration of BCG complications in infants is necessary, especially in regions with a high prevalence of IEI such as UAE.

It is important to realize that leucocyte adhesion deficiencies are rarely considered in clinical practices, and are infrequently reported in the literature. They go underdiagnosed, mainly due to the infrequent genetic testing (51).

Table 3 compares the clinical findings of the studied toddler with a few of the previously reported children. Typically, these individuals show symptoms and are diagnosed in early infancy. Bleeding and granulocytosis are consistent findings. Infections are frequent and span a wide-spectrum of pathogens (bacterial and fungal), including *Pneumocystis jirovecii, Acinetobacter baumannii*, and *Salmonella species* (Table 3).

## Data Availability Statement

The original contributions presented in the study are included in the article/Supplementary Material, further inquiries can be directed to the corresponding author/s.

## Ethics Statement

The studies involving human participants were reviewed and approved by Tawam Human Research Ethics Committee. Written informed consent to participate in this study was provided by the participants' legal guardian/next of kin. Written informed consent was obtained from the minor(s)' legal guardian/next of kin for the publication of any potentially identifiable images or data included in this article.

## Author Contributions

A-KS, RV, AY, SA-H, and LK: conceived, designed, and structured the report. RV: performed the variant analysis. A-KS, AY, AAA, HA, and AA: analyzed and interpreted the clinical data. AE: analyzed and interpreted the pathologic data. A-KS, AY, RV, and LK: wrote the original draft. A-KS, RV, and AY: reviewed and edited the paper. All authors contributed to the article and approved the submitted version.

## Conflict of Interest

The authors declare that the research was conducted in the absence of any commercial or financial relationships that could be construed as a potential conflict of interest.

## Publisher's Note

All claims expressed in this article are solely those of the authors and do not necessarily represent those of their affiliated organizations, or those of the publisher, the editors and the reviewers. Any product that may be evaluated in this article, or claim that may be made by its manufacturer, is not guaranteed or endorsed by the publisher.

## Footnotes

^1^Available online at: https://gnomad.broadinstitute.org/gene/ENSG00000149781?dataset=gnomad_r2_1 (accessed June 19, 2021).
