# Supplementary material for: Case Report: A Case of Leukocyte Adhesion Deficiency, Type III Presenting With Impaired Platelet Function, Lymphocytosis and Granulocytosis
Source: Front Pediatr. 2021 Aug 16;9:713921. doi: 10.3389/fped.2021.713921 (PMC8415448; doi:10.3389/fped.2021.713921)
Supplement: Supplementary file 1 [file Data_Sheet_1.PDF]

A case of leukocyte adhesion deficiency, type III presenting with impaired platelet function, lymphocytosis and granulocytosis

Amal M. Yahya[1], Asia A. AlMulla[2], Haydar J. AlRufaye[2], Ahmed Al Dhaheri[1], Abdulghani S. Elomami[3], Suleiman Al-Hammadi[4,5], Lalitha Kailas[6], Ranjit Vijayan[7,*], Abdul-Kader Souid[5,*]

Departments of Pediatrics[1], Hematology-Oncology[2], and Pathology[3], Tawam Hospital, Al Ain, Abu Dhabi, UAE

[4] College of Medicine, Mohamed Bin Rashid University of Medicine and Health Sciences, Dubai, UAE

[5] Department of Pediatrics, College of Medicine and Health Sciences, United Arab Emirates University Al Ain, UAE

[6] Department of Pediatrics, Sree Gokulam Medical College, Venjaramoodu, Trivandrum, Kerala, India

[7] Department of Biology, College of Science, United Arab Emirates University Al Ain, UAE

* *Correspondences*: Ranjit Vijayan, ranjit.v@uaeu.ac.ae *and* Abdul-Kader Souid, asouid@uaeu.ac.ae.

SUPPLEMENTARY INFORMATION

Analysis of the patient's platelet receptors by flow cytometry. *Left upper panel*: Acquisition of the platelet-sized events. *Remaining flow cytometry panels*: Platelet receptors were specifically identified by staining with anti-human CD61 FITC (fluorescein isothiocyanate; integrin β3), CD41 PE (R-phycoerythrin; integrin α_{IIb}), CD42a PerCP (peridinin-chlorophyll-protein Complex Conjugate; GPIX), and CD42b APC (allophycocyanin conjugate; GPlb-α). Results of each set is also shown.

FACSDiva Version 6.1.2

PLATELETS RECEPTORS ANALYSIS

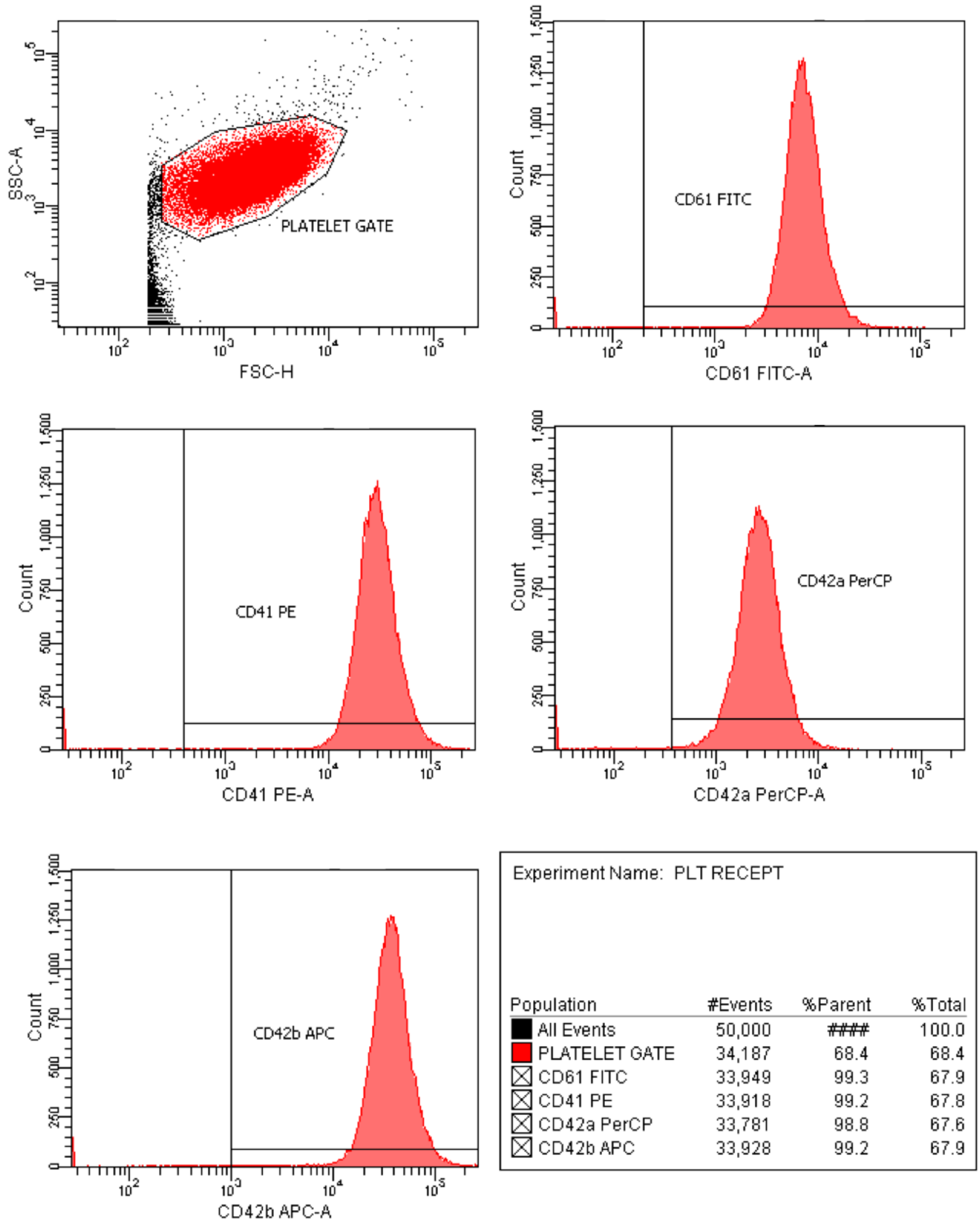

Experiment Name: PLT RECEPT

Population	#Events	%Parent	%Total
All Events	50,000	####	100.0
PLATELET GATE	34,187	68.4	68.4
CD61 FITC	33,949	99.3	67.9
CD41 PE	33,918	99.2	67.8
CD42a PerCP	33,781	98.8	67.6
CD42b APC	33,928	99.2	67.9

Expression of the patient's leukocyte receptors, assessed by flow cytometry. Sorted data from a control sample (Control 1-3) and a patient sample (Tubes 1-3) are shown. Cell size assessed by SSC-A (side scatter area) and FSC-A (forward scatter area). Leukocyte receptors were specifically identified by staining with anti-human CD18 (integrin β_2) FITC-A (Fluorescein isothiocyanate), CD11b (integrin, alpha M) APC-A (allophycocyanin conjugate), CD11a (integrin, alpha L) FITC-A, and CD11c (integrin, alpha X) APC-A, as shown. A summary of each set of results is also shown.

FACSDiva Version 6.1.3

BD FACS Canto, SN:V96101207

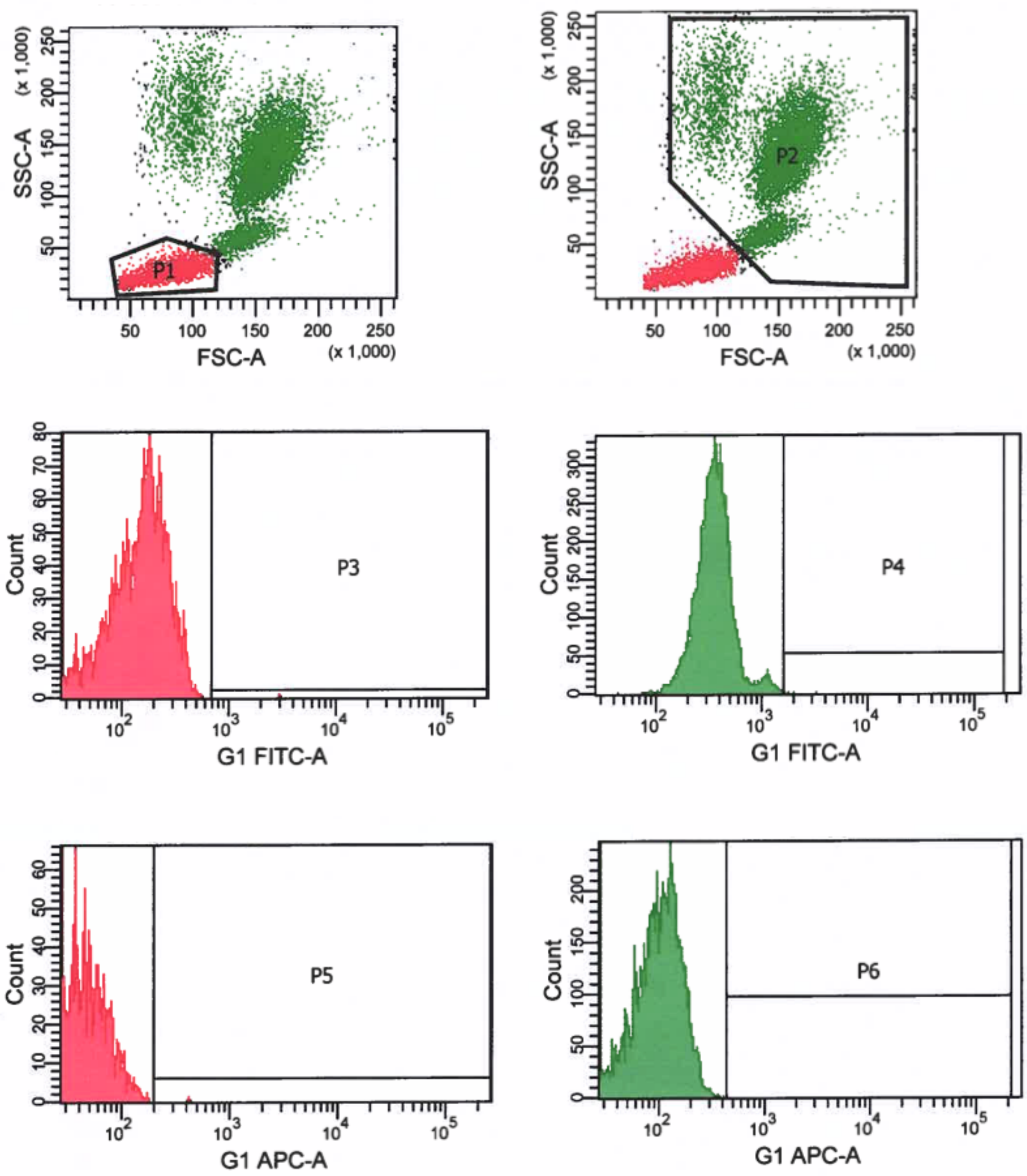

Tube: Control 1

Population	#Events	%Parent	%Total
All Events	10,000	####	100.0
P1	2,661	26.6	26.6
P3	1	0.0	0.0
P5	1	0.0	0.0
P2	7,191	71.9	71.9
P4	6	0.1	0.1
P6	1	0.0	0.0

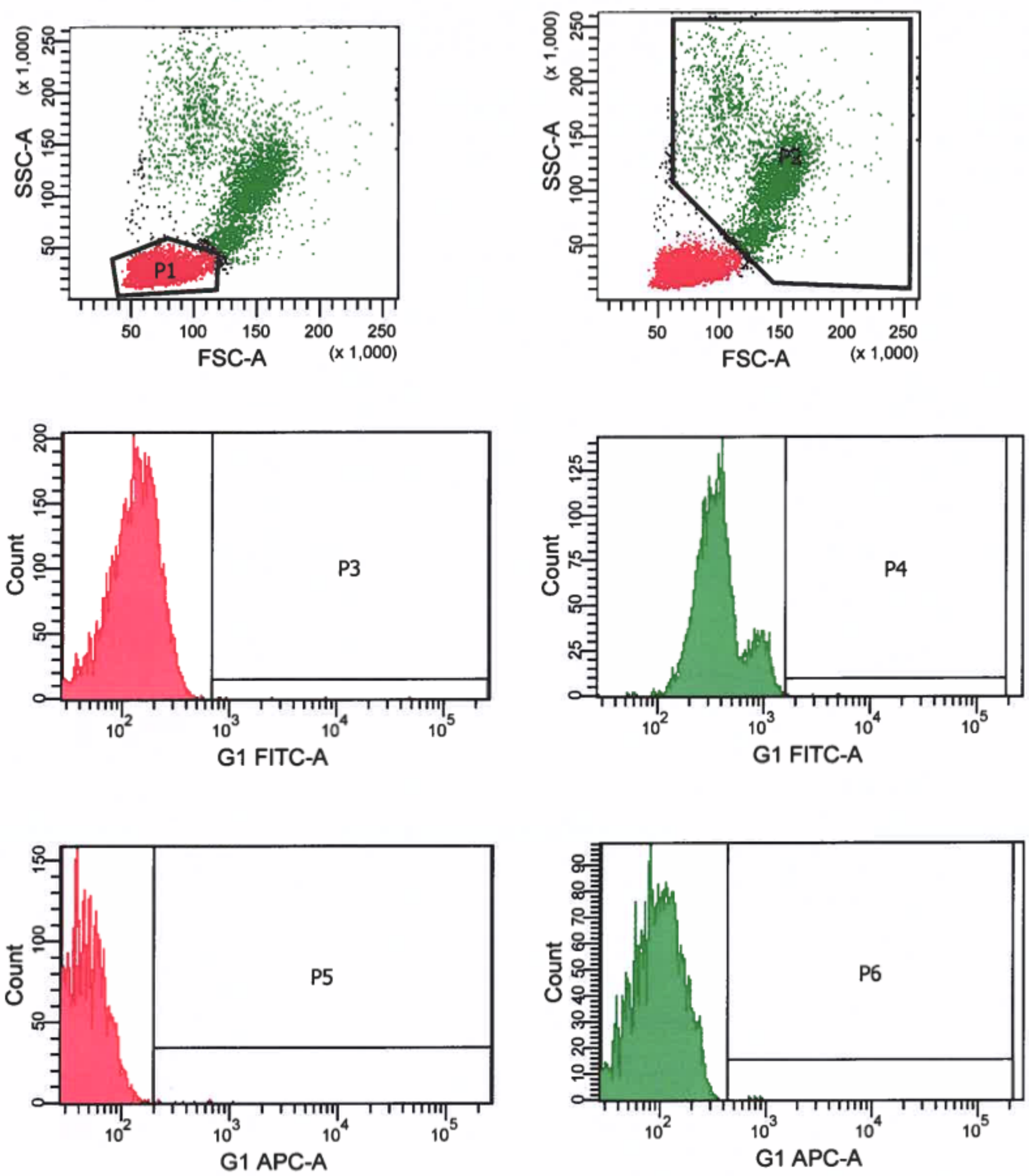

Tube: 1

Population	#Events	%Parent	%Total
All Events	10,000	####	100.0
P1	6,538	65.4	65.4
P3	6	0.1	0.1
P5	11	0.2	0.1
P2	3,299	33.0	33.0
P4	5	0.2	0.0
P6	3	0.1	0.0

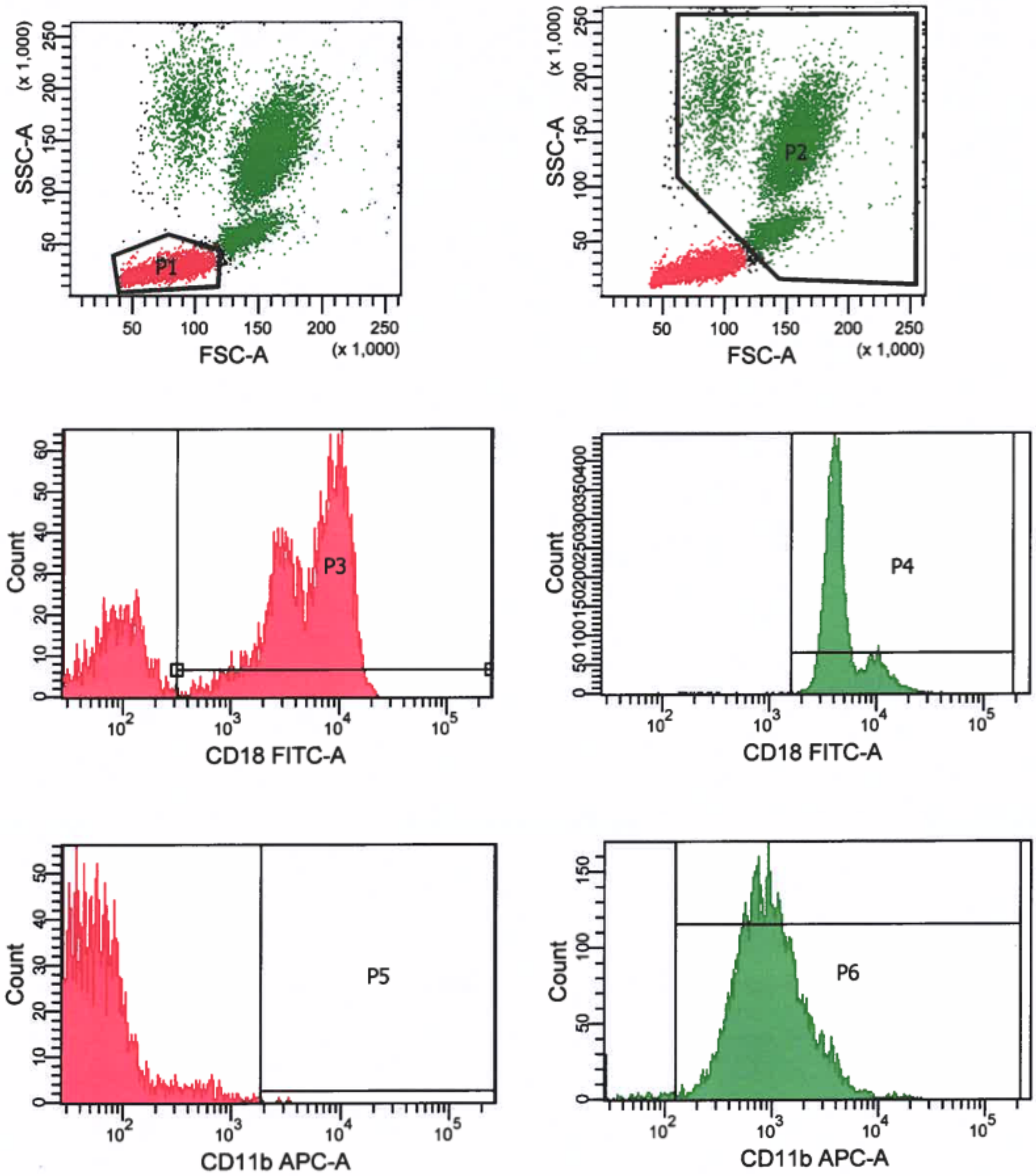

Tube: Control 2

Population	#Events	%Parent	%Total
All Events	10,000	####	100.0
P1	3,205	32.0	32.0
P3	2,336	72.9	23.4
P5	3	0.1	0.0
P2	6,649	66.5	66.5
P4	6,621	99.6	66.2
P6	6,540	98.4	65.4

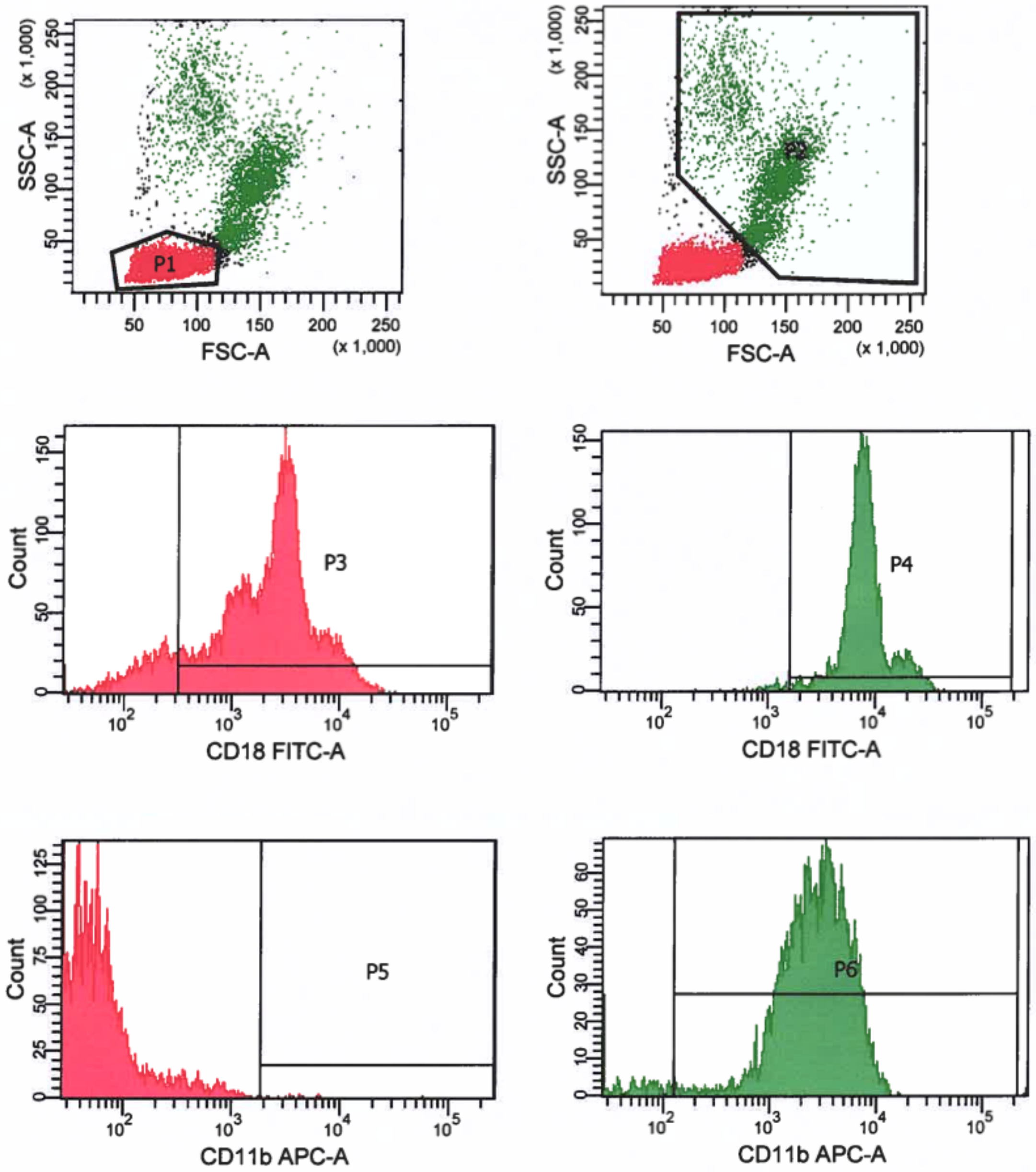

Tube: 2

Population	#Events	%Parent	%Total
All Events	10,000	####	100.0
P1	6,688	66.9	66.9
P3	5,853	87.5	58.5
P5	16	0.2	0.2
P2	3,127	31.3	31.3
P4	3,065	98.0	30.6
P6	3,006	96.1	30.1

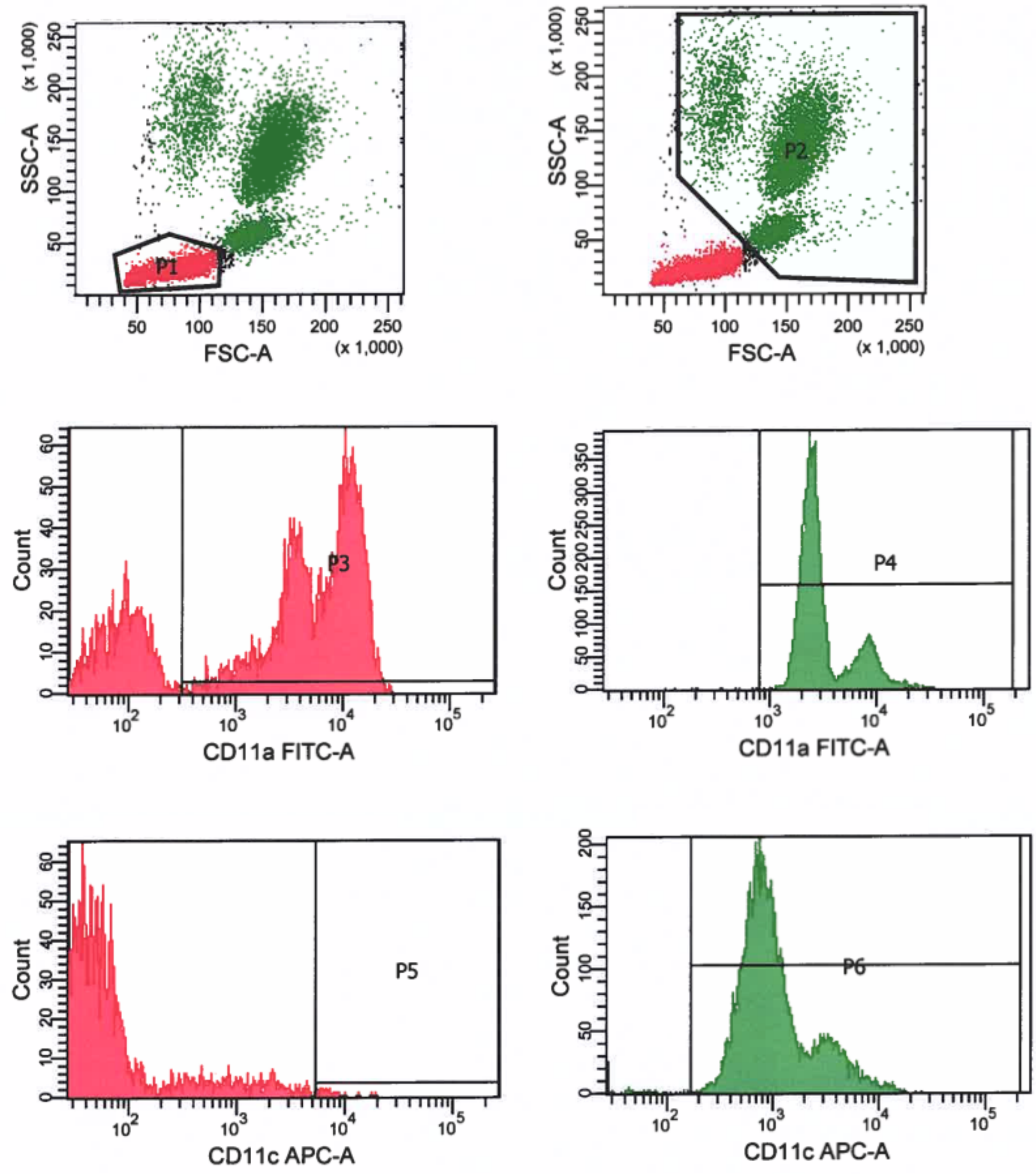

Tube: Control 3

Population	#Events	%Parent	%Total
All Events	10,000	####	100.0
P1	3,268	32.7	32.7
P3	2,307	70.6	23.1
P5	22	0.7	0.2
P2	6,567	65.7	65.7
P4	6,555	99.8	65.6
P6	6,495	98.9	65.0

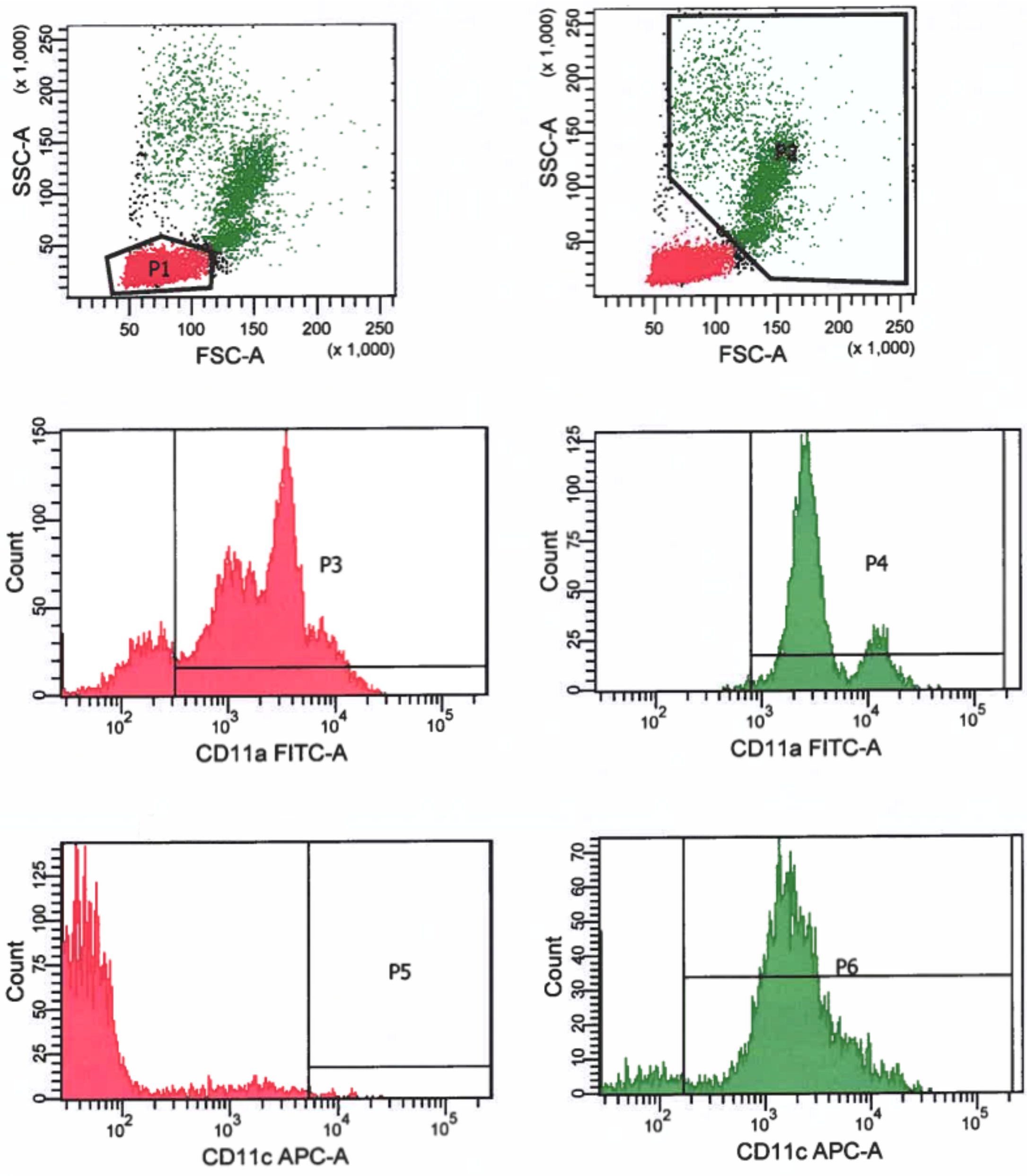

Tube: 3

Population	#Events	%Parent	%Total
All Events	10,000	####	100.0
P1	6,873	68.7	68.7
P3	5,778	84.1	57.8
P5	48	0.7	0.5
P2	2,950	29.5	29.5
P4	2,926	99.2	29.3
P6	2,711	91.9	27.1
